# Development and validation of a clinicoradiomic nomogram to assess the HER2 status of patients with invasive ductal carcinoma

**DOI:** 10.1186/s12885-022-09967-6

**Published:** 2022-08-10

**Authors:** Aqiao Xu, Xiufeng Chu, Shengjian Zhang, Jing Zheng, Dabao Shi, Shasha Lv, Feng Li, Xiaobo Weng

**Affiliations:** 1 Department of Radiology, The Central Hospital Affiliated to Shaoxing University (Shaoxing Central Hospital), Shaoxing, 312030 China; 2 Department of Surgical, The Central Hospital Affiliated to Shaoxing University (Shaoxing Central Hospital), Shaoxing, 312030 China; 3 Department of Radiology, Fudan University Shanghai Cancer Center, Shanghai, 200032 China; 4 Department of Research Collaboration, R&D center, Beijing Deepwise & League of PHD Technology Co., Ltd, Beijing, 100080 P.R. China

**Keywords:** Breast carcinoma, Multiparametric magnetic resonance imaging, Nomograms, HER2, Radiomics

## Abstract

**Background:**

The determination of HER2 expression status contributes significantly to HER2-targeted therapy in breast carcinoma. However, an economical, efficient, and non-invasive assessment of HER2 is lacking. We aimed to develop a clinicoradiomic nomogram based on radiomics scores extracted from multiparametric MRI (mpMRI, including ADC-map, T2W1, DCE-T1WI) and clinical risk factors to assess HER2 status.

**Methods:**

We retrospectively collected 214 patients with pathologically confirmed invasive ductal carcinoma between January 2018 to March 2021 from Fudan University Shanghai Cancer Center, and randomly divided this cohort into training set (*n* = 128, 42 HER2-positive and 86 HER2-negative cases) and validation set (*n* = 86, 28 HER2-positive and 58 HER2-negative cases) at a ratio of 6:4. The original and transformed pretherapy mpMRI images were treated by semi-automated segmentation and manual modification on the DeepWise scientific research platform v1.6 (http://keyan.deepwise.com/), then radiomics feature extraction was implemented with PyRadiomics library. Recursive feature elimination (RFE) based on logistic regression (LR) and LASSO regression were adpoted to identify optimal features before modeling. LR, Linear Discriminant Analysis (LDA), support vector machine (SVM), random forest (RF), naive Bayesian (NB) and XGBoost (XGB) algorithms were used to construct the radiomics signatures. Independent clinical predictors were identified through univariate logistic analysis (age, tumor location, ki-67 index, histological grade, and lymph node metastasis). Then, the radiomics signature with the best diagnostic performance (Rad score) was further combined with significant clinical risk factors to develop a clinicoradiomic model (nomogram) using multivariate logistic regression. The discriminative power of the constructed models were evaluated by AUC, DeLong test, calibration curve, and decision curve analysis (DCA).

**Results:**

70 (32.71%) of the enrolled 214 cases were HER2-positive, while 144 (67.29%) were HER2-negative. Eleven best radiomics features were retained to develop 6 radiomcis classifiers in which RF classifier showed the highest AUC of 0.887 (95%CI: 0.827–0.947) in the training set and acheived the AUC of 0.840 (95%CI: 0.758–0.922) in the validation set. A nomogram that incorporated the Rad score with two selected clinical factors (Ki-67 index and histological grade) was constructed and yielded better discrimination compared with Rad score (*p* = 0.374, Delong test), with an AUC of 0.945 (95%CI: 0.904–0.987) in the training set and 0.868 (95%CI: 0.789–0.948; *p* = 0.123) in the validation set. Moreover, calibration with the *p*-value of 0.732 using Hosmer–Lemeshow test demonstrated good agreement, and the DCA verified the benefits of the nomogram.

**Conclusion:**

Post largescale validation, the clinicoradiomic nomogram may have the potential to be used as a non-invasive tool for determination of HER2 expression status in clinical HER2-targeted therapy prediction.

**Supplementary Information:**

The online version contains supplementary material available at 10.1186/s12885-022-09967-6.

## Introduction

Breast carcinoma is the most widespread and lethal tumor among women [1]. The most common histological type is invasive ductal carcinoma (IDC), which accounts for roughly 80% of all breast carcinomas, with 20% – 30% expressing human epidermal growth factor receptor 2 (HER2) positivity [2]. Numerous studies substantiated the association of HER2 gene overexpression with the division, proliferation, and nourishment of new vasculature of breast carcinoma cells. Overexpression of HER2 is significantly correlated with high aggressiveness and poor prognosis in breast carcinoma patients [3–6]. Consequently, advancement in HER2-targeted therapy was considered a prominent breakthrough in breast carcinoma treatment [7, 8]. Therefore, determining the HER2 expression status in breast carcinoma patients is an essential precondition to identifying potential candidates for treatment decisions. The immunohistochemistry (IHC) method for testing HER-2 expression is a standard procedure in our laboratory and a part of our pathology reports in invasive breast carcinomas, and FISH is the subsequent and gold method in equivocal (2 +) IHC cases [9]. These two methods are more time-consuming, and takes 5–12 days after specimens are provided. FISH is difficult to apply, and requires trained technicians. It is more expensive since it requires test kits and a special microscope, and stained preparations cannot be archived [10]. Radiomics allows inference of tumoral molecular status from medical image-derived features, and it allows the study of the tumoral heterogeneity both spatially and over time; radiomics have the potential to enable spatio-longitudinal monitoring of tumor biology before and during treatment.

Existing literature has recently documented a correlation between MRI image expression and breast carcinoma HER2 expression level [11–15]. Song et al. [11] reported that texture features derived from kinetic parameter maps, calculated based on breast DCE-T1W1, can be used as imaging biomarkers to distinguish HER2-positive and HER2-negative breast cancer. The best model with features extracted from the slope of signal intensity (SIslope) map yielded an AUC of 0.79 in the test set. Another radiomic study by Li et al. [12] selected ER status and radiomics features in DWI images to establish the nomogram, which yielded good discrimination (AUC: 0.883/0.848) and calibration. The radiomics nomogram showed favorable performance for evaluating HER2 status in breast cancer. To the best of our knowledge, the HER2 express status in breast carcinoma could be better predicted by radiomics signature established from the mpMRI compared with single-parametric signature. Zhou et al. found the prediction ability of the developed SVM model in HER2 status of breast carcinoma patients based on T2WI, DCE-T1W1, and a combination of the two sequences. The multiparameter-related model manifested the best efficiency in forecasting the HER2 status with an AUC of 0.86 and 0.81 in the learning and test cohorts, respectively [16]. However, no published study has discussed the MRI combining with clinical pathological risk factors, such as Ki-67 index, histological grade, and lymph node metastasis, in predicting HER2 expression, and no published study has applied clinicoradiomic models (nomogram) using multiple machine learning algorithms in predicting HER2 expression in invasive ductal carcinoma.

This paper aims to investigate and compare the performance of radiomics features from different types of MR images (fat-suppressed T2-weighted images (T2WI), diffusion coefficient map (ADC-map), and dynamic contrast-enhanced T1-weighted images (DCE-T1WI)) and the performance of their fusions using different machine learning algorithms. In addition, a nomogram incorporating the mpMRI-based radiomics signature and clinical predictors was developed to improve the discriminative and interpretable ability of the classifier for HER2 status prediction.

## Materials and methods

### Study population

**Fig. 1:**
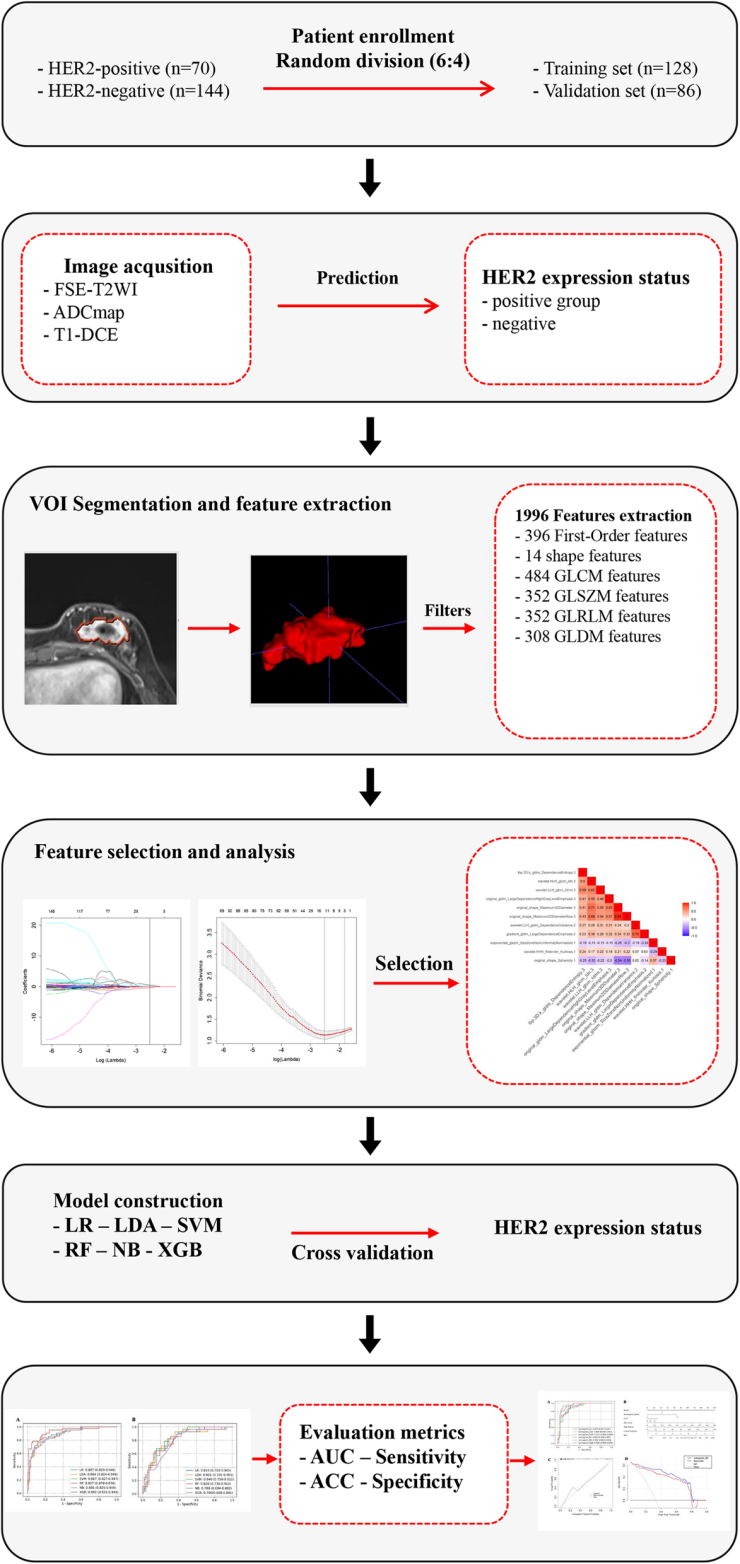
The flowchart of this study

The Fudan University Shanghai Cancer Center and Shaoxing Central Hospital Ethical Committee approved this study protocol, and the requirement for written informed consent was waived. Between January 2018 and March 2021, we retrospectively collected breast tumor cases confirmed by clinical examination and diagnosed by ultrasound examination in our center. Inclusion criteria: (I) the patient had proven invasive breast ductal carcinoma of no special type (IDC-NST), as determined by histology; (II) complete breast mpMRI data and pathological data, HER2 2 + status verified by FISH; Exclusion criteria: (I) pregnant or lactating females, or a plan to get pregnant within six months; (II) prosthesis implantation; (III) history of breast surgery that might affect imaging diagnosis. A total of 302 consecutive patients from Fudan University Shanghai Cancer Center were eligible. However, poor image quality or incomplete lesion presentation in 25 cases, neoadjuvant therapy in 46 cases, and a history of breast carcinoma or bilateral breast carcinoma in 17 cases were excluded. In the case of multicentric lesions, the largest major lesion of them was selected to enter the group. Patient demographic data, including age, tumor location, menopause status, and a family history of breast carcinoma in first-degree relatives, were retrieved from the Electronic Medical Record System. Pathological data were tumor pathological type and histological grade, the expression status of HER2, Ki-67 index, and lymph node metastasis. The final cohort comprised of 214 patients and was randomly split with a ratio of 6:4 [17, 18], to create the training set (*n* = 128, 42 HER2-positive and 86 HER2-negative cases) and validation set (*n* = 86, 28 HER2-positive and 58 HER2-negative cases), respectively for subsequent analyses. Figure 1 illustrates the flowchart of this study.

### Imaging examination

For all patients the breast MRI examination was performed on the Aurora Dedicated Breast MRI System and a dedicated phased-array coil. The patients were scanned in the prone position to allow both mammary glands in the concave hole of the phased-array coil with a natural overhanging effect. All these sequences were obtained back-to-back in one imaging session. Imaging protocols were as follows: (1) transverse T2WI with fat-suppression (FS-T2WI, TR 6680 ms, TE 68 ms), with the layer thickness and spacing of 3 mm and 1 mm, respectively; (2) transverse DWI (TR8 400 ms, TE 84 ms), DWI signal intensity at *b* = 0 s/${\mathrm{mm}}^{2}$, *b* = 1,000 s/${\mathrm{mm}}^{2}$, with the layer thickness and spacing of 4 mm, and 6 mm, respectively, and a DWI-derived ADC map; (3) 3D-DCE, transverse T1WI with fat and water suppression (TR 5 ms, TE 29 ms) was selected, with a layer thickness of 1.1 mm and a layer spacing of 0, FOV 360 mm × 360 mm, matrix 360 × 360 × 128. The number of scanned layers in a single phase was 160. Phase I mask scan was done prior to the contrast enhancement scan. In the contrast scan, Gd-DTPA (Bayer Medical Care Ltd) was administered intravenously at a dose of 0.2 mmol/kg and a flow rate of 2.0 mL/s. After injection, contrast images were obtained in 5 phases in a row, with a scan time of 120 s per phase.

### Image analysis

The breast MRI data of all sequences were imported as a DICOM file into the DeepWise scientific research platform v1.6 (http://keyan.deepwise.com/). Two radiologists with more than 10-year experience in breast imaging diagnosis semi-automatically segmented lesions layer-by-layer and analyzed after merging them into the three-dimensional region of interests (3D ROIs, VOIs). After carefully scrutinizing the tumor region, disagreements were resolved by consensus-based discussion or decided by superior physicians. The 3rd sequence during the dynamic enhancement course was selected after about 240 s of injecting contrast medium. At this point, malignant lesions generally show a peak enhancement to present a clear contrast with the surrounding normal breast parenchyma, which is conducive to more accurate VOI delineation and feature extraction. The selected VOI should conform to the following criteria: (1) Include cystic lesion, necrosis, and halo-sign; (2) Invasion of surrounding structures: areas connected to VOI and have the same enhancement pattern with the VOI; (3) The lesion VOI < 5 ${\mathrm{mm}}^{3}$ is waived. The VOIs of T1-DCE were registered and applied to the other two sequences, and the image slicers and orientation were precisely matched between T2WI and ADC-map. Supplemental Figure S1 provided the process of segmentation and VOI selection of two typical HER2-positive and HER2-negative cases. Then, B-spline interpolation was carried out to standard the image into the same spatial resolution (1 mm × 1 mm × 1 mm)[19–21] and followed by the absolute gray-level discretization with fixed bin size (FBS) set to 5 as previous studies suggested [20–23]. In particular, the processes of image interpolation and gray-level discretization were both aligned to IBSI [21].

### Radiomics feature extraction and screening

To emphasize the imaging characteristics, image filters such as log (Laplacian of Gaussian), gradient, lbp-2d/3d, and four common point-level transforms were applied prior to feature extraction. In addition, wavelet decomposition was applied at each channel for images to obtain all possible combinations in high-pass or low-pass filters (LLH, LHL, LHH, HLL, HLH, HHL, HHH, LLL). For original and other-transformed images, first-order, shape, and texture features were extracted, respectively, and implemented with the open-source PyRadiomics library (https://github.com/Radiomics/pyradiomics). Subsequently, Z-score transformation was used to normalize the feature distribution in the training set, and the data in the validation set were then standardized by the same calculated parameters to avoid data leakage. The implementation of feature extraction and standardization was compliant with Imaging Biomarker Standardization Initiative (IBSI) [21]. For each sequence, we initially extracted 1906 radiomics features from VOIs, including 396 first-order features, 14 shape features (including three 2D features), and 1496 texture features. A suffix was added for the different series (i.e., ‘1’ represents ADC-map, ‘2’ represents T2WI, ‘3’ represents DCE-T1WI). Intra-class correlation coefficient (ICC) was calculated to evaluate the intra- and inter-observer reproducibility of radiomics features. Initially, reader 1 and reader 2 completed the VOI segmentation and radiomics feature extraction on 30 randomly selected patients’ mpMRI data. Then, Reader 1 repeated the same procedure 1 month later. Intra- and inter-observer ICC variabilities were studied using the one-way random single measures ICC (ICC(1,1)) and two-way random single measures ICC (ICC(2,1)), respectively. And features with intra- or inter-observer ICC values lower than 0.75 were discarded before subsequent feature selection. Moreover, we applied Pearson’s correlation with a threshold of 0.9 in training set to minimize the potential collinearity of variables.

### Model establishment and evaluation

Given the extracted high-throughput radiomics features, dimension reduction of the retained features was further performed with the algorithms of recursive feature elimination (RFE) and LASSO. These feature selection algorithms were used in this study because of efficiency and popularity. To achieve high and robust performance of the developed classifiers, six machine learning algorithms, Logistic Regression (LR), Linear Discriminant Analysis (LDA), Support Vector Machine (SVM), Random Forest (RF), Naive Bayesian (NB), XGBoost (XGB), were used to classifier construction. The reason for selecting and comparing these methods in this study was that they were common classifiers in the related study for breast in previous studies, such as undergoing mastectomy prediction [24], breast cancer prediction [25], axillary lymph node metastasis [26]. To avoid over-fitting in the modeling process, the hyper-parameter searching for optimal classifiers were completed by grid-search method using the tenfold cross-validation repeatedly. After the completion of radiomics classifiers, multivariate logistic regression analysis and backward stepwise regression analysis based on Akaike Information Criterion (AIC) were devised to establish a clinicoradiomic model (nomogram) incorporating significant clinical predictors and radiomics classifiers. Thus, we developed the visual nomogram of the clinicoradiomic model to calculate the probability of HER2-positive breast cancer.

### Pathological analysis

Two pathologists with more than 15 years of expertise identified the histological type, histological grading, and immunohistochemical analysis. The histological type of breast carcinoma was defined according to the World Health Organization classification. The Elston–Ellis System was followed to estimate the tumor histological grade (Elston and Ellis 1991). IHC or FISH determined the HER2 status based on the clinical use instructions for HER2 experiment in breast carcinoma presented by the American Society of Clinical Oncology (ASCO)/College of American Pathologists (CAP) [27], IHC scores of 3 + and 0 or 1 + were considered positive, and negative, respectively, while an IHC score of 2 + of HER2 was regarded as indeterminate. To determine gene amplification, researchers used fluorescence in situ hybridization (FISH), and the ratio ≥ 2.0 was judged positive for HER2 [27, 28].

### Statistical methods

Statistical analysis was conducted on R statistical software v3.6.1 (http://www.Rproject.org). Student’s t-test and Chi-square test were, respectively, used for continuous and categorical data with normal distribution, and the Mann–Whitney U test was applied for data with non-normal distribution. All tests were two-tailed, and *p* < 0.05 was considered statistically significant. The receiver operating characteristic (ROC) curve is defined for evaluating the performance of binary classifiers and is produced by calculating the true positive rate against the false positive rate for a binary classifier at a set of different thresholds. And we used Youden’s index (YI) to calculate the optimal threshold, which gives equal weight to specificity and sensitivity. The R package “glmnet” statistical software (R Foundation) was used to perform the modeling process of classifiers. “PROC” R package was mainly used in the ROC curve analysis. In addition, DeLong test was used to compare the performance of two different models. Calibration curves of the nomogram and Hosmer–Lemeshow test were used to validate the agreement between prediction and observation in validation set. Furthermore, we performed decision curve analysis (DCA) to visualize the net benefit for clinical decisions.

## Results

### Clinical data and pathological diagnosis results

The detailed clinical and histopathologic characteristics of patients with IDC were summarized in Table 1. Of the 214 eligible cases, the age ranged from 16 to 86 years, with an average age of 50.77 ± 10.29 years. This cohort encompassed HER2-positive in 70 (32.71%, 70/214) cases and HER2-negative in 144 (67.29%,144/214) cases. There was no significant difference between the two groups in all clinical features (p > 0.05).

**Table 1:** Clinical and histopathologic characteristics of IDC patients

Characteristics	Train Cohort		*P* value	Validation Cohort	
	HER2 + (*n* = 42)	HER2-(*n* = 86)		HER2 + (*n* = 28)	HER2-(*n* = 58)
Ki-67	47.98 ± 20.51	33.35 ± 23.04	0.94	46.25 ± 23.16	34.62 ± 27.30
Patient age			0.166		
< 35 (youth)		1(1.16)		3(10.71)	1(1.72)
30–50 (middle-aged)	26(61.90)	60(69.77)		17(60.71)	36(62.07)
> 50 (menopause)	16(38.10)	25(29.07)		8(28.57)	21(36.21)
Location = Central District			0.243		
No	38(90.48)	73(84.88)		24(85.71)	55(94.83)
Yes	4(9.52)	13(15.12)		4(14.29)	3(5.17)
Position = upper-right quadrant			0.209		
No	32(76.19)	63(73.26)		18(64.29)	39(67.24)
Yes	10(23.81)	23(26.74)		10(35.71)	19(32.76)
Position = Lower-right quadrant			0.5		
No	34(80.95)	75(87.21)		24(85.71)	52(89.66)
Yes	8(19.05)	11(12.79)		4(14.29)	6(10.34)
Position = Upper left quadrant			0.463		
No	25(59.52)	52(60.47)		20(71.43)	36(62.07)
Yes	17(40.48)	34(39.53)		8(28.57)	22(37.93)
Position = Lower left quadrant			0.05		
No	37(88.10)	78(90.70)		22(78.57)	47(81.03)
Yes	5(11.90)	8(9.30)		6(21.43)	11(18.97)
Histologicalgrades			0.256		
Stage I	1(2.38)	5(5.81)			9(15.52)
Stage II	11(26.19)	56(65.12)		12(42.86)	32(55.17)
Stage III	30(71.43)	25(29.07)		16(57.14)	17(29.31)
Lymph node metastasis			0.534		
0	24(57.14)	51(59.30)		13(46.43)	29(50.00)
1	9(21.43)	21(24.42)		7(25.00)	19(32.76)
2	5(11.90)	9(10.47)		6(21.43)	6(10.34)
3	4(9.52)	5(5.81)		2(7.14)	4(6.90)

### Predictive efficiency of the radiomics signatures

**Fig. 2:**
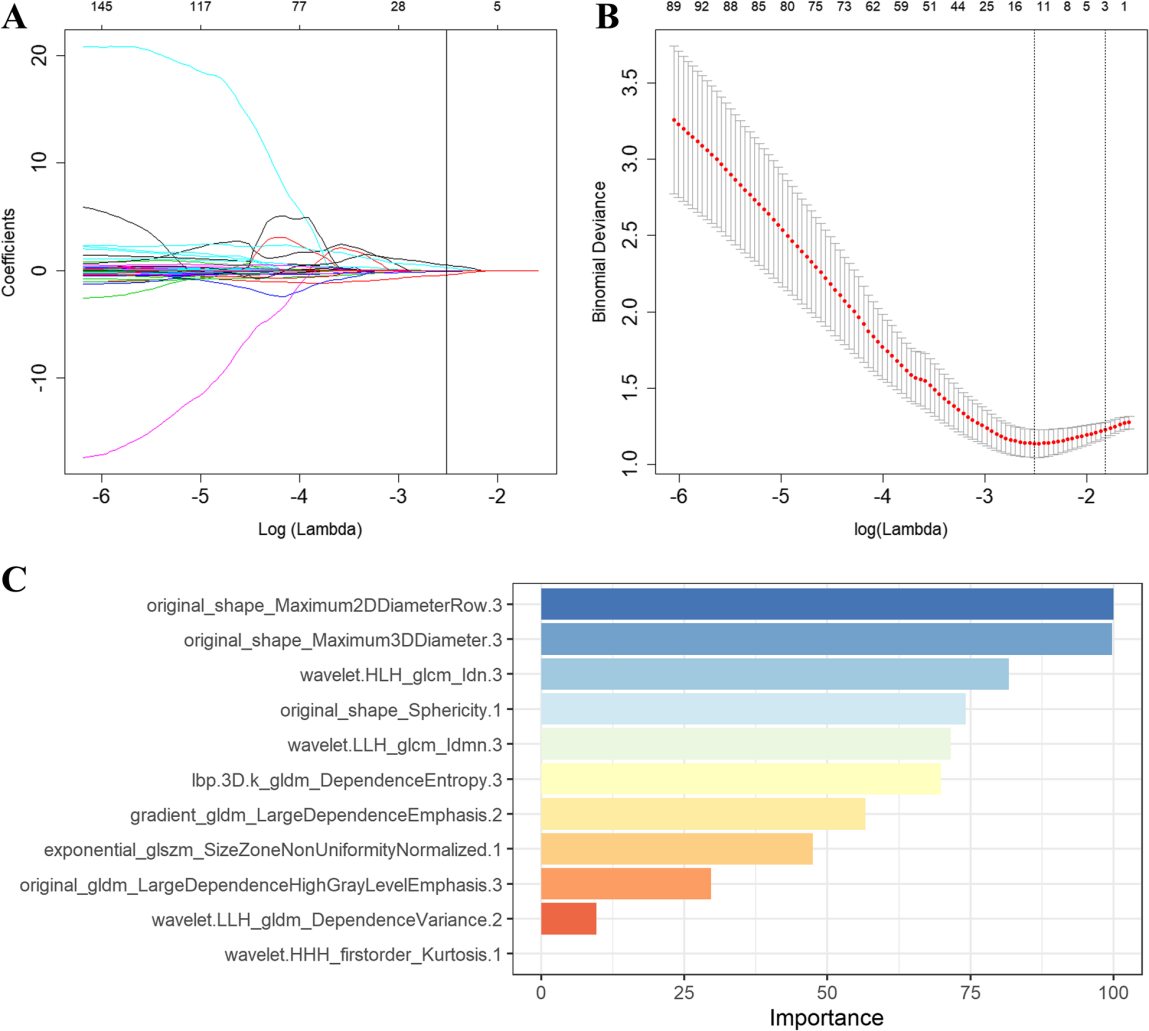
**A** Feature coefficients corresponding to the value of parameter λ. Each curve represents the change trajectory of each independent variable. **B** The most valuable features were screened out by tuning λ using LASSO via minimum binomial deviation. The dotted vertical line represents the optimal log (λ) value. **C** The selected 11 radiomics features with the most discriminative value

**Fig. 3:**
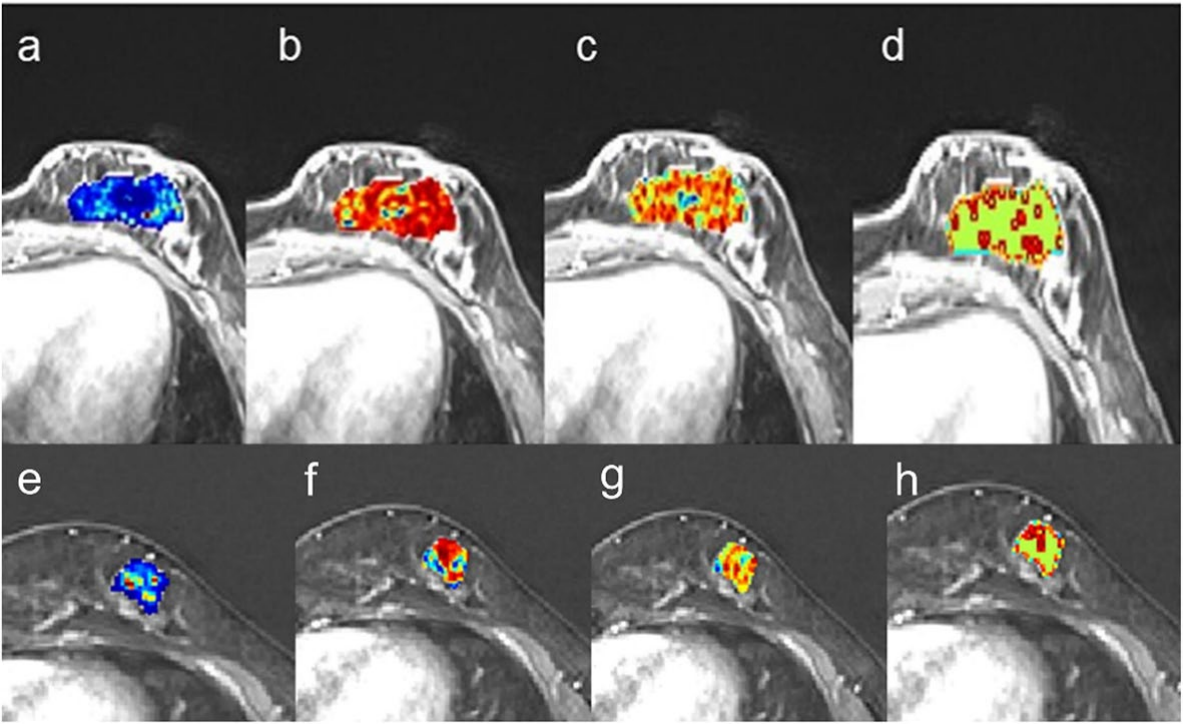
Qualitative visualizations of 4 top Radiomics features on the DCE-T1W1 sequence between HER2-positive (**a**-**d**) and HER2-negative cases (**e**–**h**). **a**,**e** Original_gldm_LargeDependenceHighGrayLevel Emphasis (**b**,**f**) wavelet-LLH_glcm_Idmn (**c**,**g**) wavelet-HLH_glcm_Idn (**d**,**h**) lbp-3D-k_gldm_DependenceEntropy

**Fig. 4:**
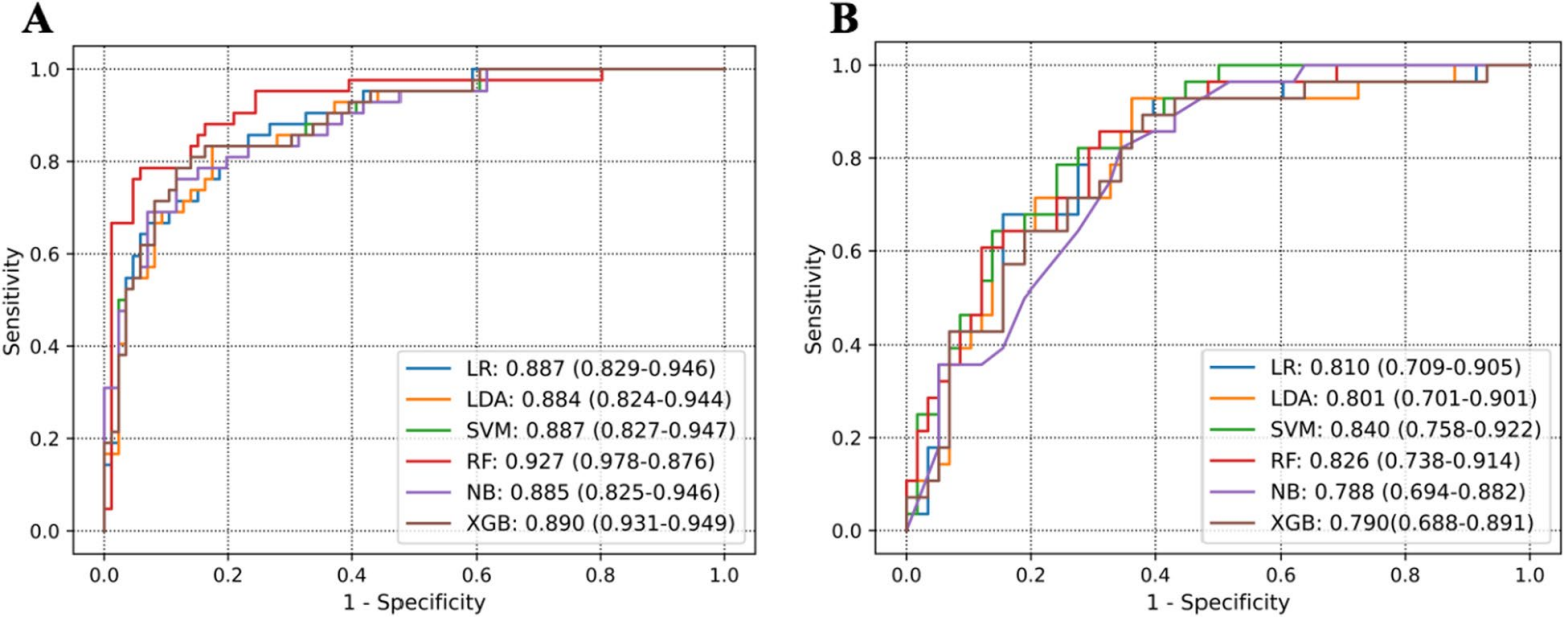
ROC curves of the six radiomics classifiers. **A** Training set. **B** Validation set

In total, 455 radiomics features were excluded through stability analysis (ICC ≤ 0.75). 345, 356, 449, and 1150 radiomic features selected from T2WI, ADC-map, DCE-T1WI, and mpMRI (including T2WI, ADC-map, DCE-T1WI) images with the |correlation coefficient|≤ 0.9 were used for next dimension reduction. The top 100 ranking of RFE remained features were further selected based on LR. Finally, 11 radiomics features were retained from the mpMRI images, T2WI (*n* = 2), ADC-map (*n* = 3), DCE-T1WI (*n* = 6), to construct the radiomics signature using the LASSO regression (Fig. 2). The relative importance of the 11 selected radiomics features for predicting HER2 expression status was shown in Fig. 2C. Figure 3 provided the qualitative visualizations of some of the top Radiomics features on the MRI sequences for a HER2-negative case and a HER2-positive case. We further verified that there was no statistically significant difference between the final selected radiomics features in the training and validation sets (Supplemental Table S1). For three sequences, the optimal performance was reported by the RF classifier (Rad score, Fig. 4) with the AUCs of 0.927 (95%CI: 0.876–0.978) (accuracy 0.851; sensitivity 0.881; specificity 0.837) in the training set and 0.826 (95%CI: 0.738–0.914) (accuracy 0.744; sensitivity 0.857; specificity 0.689) in the validation set (Table 2). The Rad score based on mpMRI images achieved higher AUC values than other single-parametric models (i.e., ADC-map, T2W1, and DCE-T1W1) in training (AUC: 0.816, 0.743 and 0.810) and validation sets (AUC: 0.719, 0.694 and 0.756) (Supplemental Figure S2).

**Table 2:** Performances of the six machine learning classifiers for predicting HER2 status in the validation cohort

Radiomics classifier	Accuracy	True positive rate (Sensitivity)	True negative rate (Specificity)	Threshold	AUC(95%CI)
LR	0.750	0.893	0.621	0.514	0.810 (0.709–0.905)
LDA	0.733	0.829	0.718	0.547	0.801 (0.701–0.901)
SVM	0.756	0.821	0.724	0.545	0.840 (0.758–0.922)
RF	0.744	0.857	0.689	0.546	0.826 (0.738–0.914)
NB	0.755	0.786	0.741	0.527	0.788 (0.694–0.882)
XGB	0.710	0.857	0.637	0.494	0.790 (0.688–0.891)

*LR* Logistic Regression, *LDA* Linear Discriminant Analysis, *SVM* Support Vector Machine, *RF* Random Forest, *NB* Naive Bayesian, *XGB* XGBoost

### Nomogram establishment

**Fig. 5:**
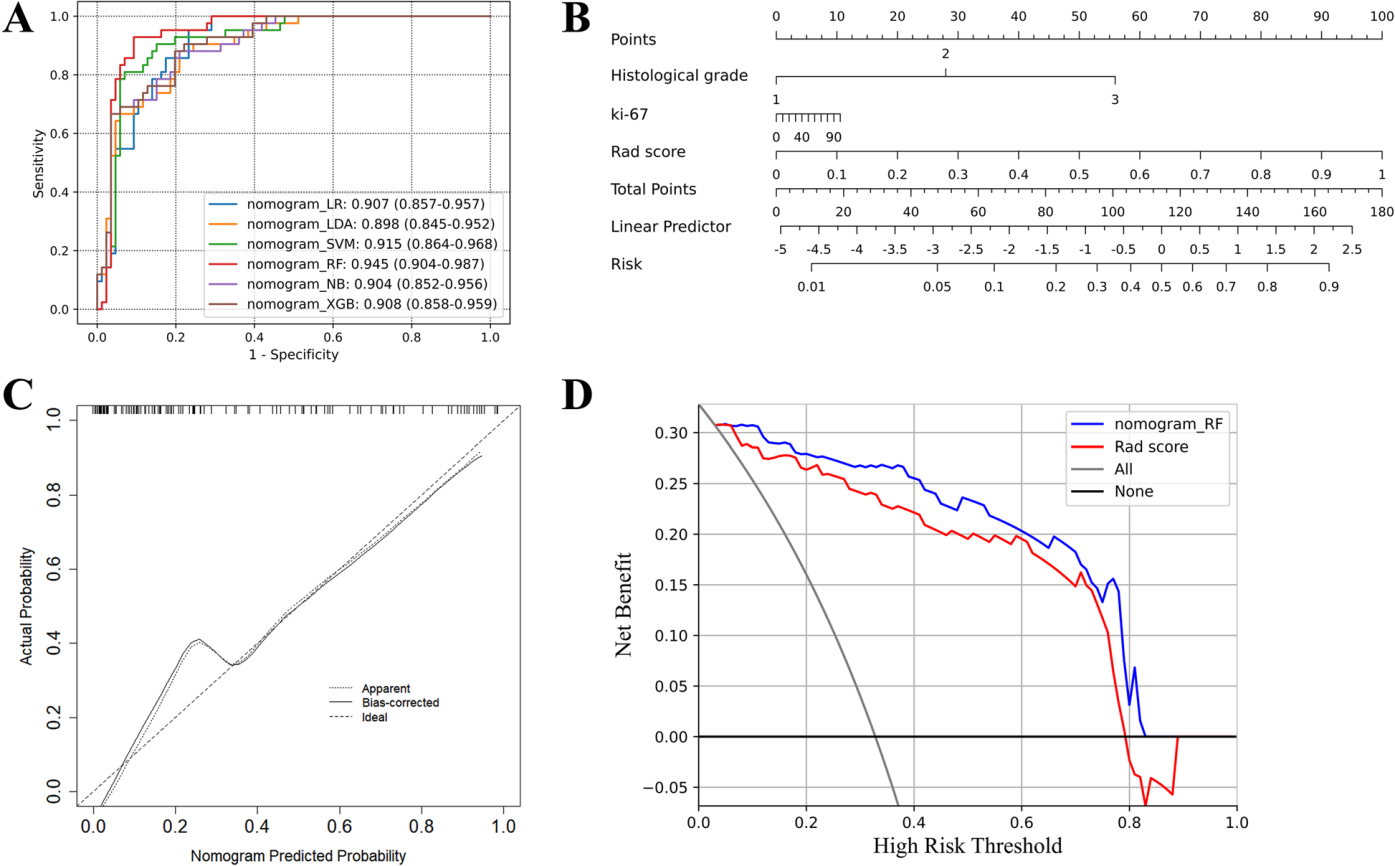
Nomogram performances for predicting HER2 status of breast carcinoma in the Training set. A. ROC curves of the six nomograms based on different radiomics classifiers. B. Nomogram_RF. C. Calibration curve of the nomogram_RF. D. Decision curves of the nomogram_RF and Rad score

The Rad score (OR = 58.909, 95%CI: 7.693 to 451.094, *p* < 0.01), histological grade (OR = 4.971, 95%CI: 2.297 to 10.757, *p* < 0.01), and ki-67 (OR = 4.435, 95%CI: 1.991 to 9.883, *p* < 0.01) were determined as independent predictors using univariate logistic regression analysis and were used to develop the nomogram (Fig. 5A, B) by multivariate logistic regression analysis (Table 3). The nomogram_RF obtained the AUC of 0.945 (95%CI: 0.904 to 0.987) in the training and 0.868 (95%CI: 0.789 to 948) in the validation cohorts. As comparison, the nomograms based on the other radiomics classifiers were constructed in the same approach (Fig. 5A). The nomogram_RF still produced the highest AUC though there was no significant added value between the radiomics classifiers and their corresponding nomograms in the training and validation sets (Supplemental Table S2). The calibration curves demonstrated that the nomogram_RF could provide an excellent calibration in the training cohort (Fig. 5C), in which the Hosmer–Lemeshow test showed a non-significant *p*-value of 0.732. The DCA curves claimed that the good application of the nomogram_RF in the clinical decisions, followed by considering the radiomics classifier alone (Fig. 5D). The performances in the validation set were shown in Supplemental Figure S3.

**Table 3:** Results of univariate and multivariate logistic regression analysis for nomogram_RF

Features	Univariate logistic regression			multivariate logistic regression	
	OR(95%CI)	*p* value		OR(95%CI)	*p* value
Location = Central	0.591(0.18–1.938)		0.39	NA	NA
Position = upper-right quadrant	0.856(0.364–2.014)		0.72	NA	NA
Position = Lower-right quadrant	1.604(0.592–4.347)		0.35	NA	NA
Position = Upper -left quadrant	1.04(0.49–2.208)		0.92	NA	NA
Position = Lower-left quadrant	1.318(0.403–4.305)		0.65	NA	NA
Histological grade	4.971(2.297–10.757)		< 0.01**	2.666 (1.003–7.085)	0.049*
Lymph node metastasis	1.133(0.768–1.673)		0.53	NA	NA
ki-67	4.435(1.991–9.883)		< 0.01**	1.012 (0.989–1.035)	0.305
Rad score	58.909(7.693–451.094)		< 0.01**	75.428(7.64–745.212)	< 0.01**

## Discussion

The present study nurtured the quantitative features from T2WI, ADC-map, and DCE-T1WI images to standardize radiomics models for the noninvasive evaluation of the HER2 status in breast carcinoma. The Rad score classifier based on the RF algorithm exhibited good efficiency in differentiating HER2-positive from HER2-negative disease, in which the AUCs, accuracy, true positive, and negative rates were 0.927 (95%CI: 0.876–0.978), 85.1%, 88.1% and 83.7% respectively, in the training cohort, and 0.826 (95%CI: 0.738–0.914), 74.4%, 85.7% and 68.9%, respectively, in the validation cohort. We then created a nomogram by combining the Rad score with the independent clinical predictors (histological grade and ki-67) with univariate and multivariate logistic regressions. The finding demonstrated that the nomogram could provide superior classification and recognition in HER2-positive patients (the AUCs of 0.945 and 0.868 in the training and validation sets). Thus, we concluded that applying radiomics features from mpMRI data incorporating machine learning algorithm contributed to the good discriminative performance in the HER2 status.

Recently, there has been a growing interest in applying quantitative imaging data to delineate intrinsic biological characteristics [29–34]. Diverse research has dissected the dependence between radiomic signatures and HER2 expression status [35–40]. Zhou et al. [35]. noninvasively evaluated the efficacy of mammography radiomics signatures in diagnosing the patients’ HER2 status with breast carcinoma, including mediolateral oblique (MLO) and cranial caudal (CC) views, with an AUC of 0.846 in the training set and an AUC of 0.74 in the testing set. Another similar study also highlighted the connection between radiomics signatures from multidetector computed breast mammography images with HER2 express status [36]. Daniele La Forgia et al. [41]. reported that radiomics combining contrast-enhanced spectral mammography performed well in predicting histological subtypes of breast cancer, with accuracies of 90.87%and 84.80% in discriminating HER2 + /HER2 − and Ki67 + /Ki67 − breast cancer, respectively. Those studies indicated that radiomics analysis was a useful analytical tool to predict HER2 status in breast cancer, which is consistent with the findings of this study.Another radiomic study by Bitencourt et al. [42].developed machine learning models (including both clinical and radiomics MRI features) to predict HER2 expression levels and pathologic response (pCR) after neoadjuvant chemotherapy in HER2 over-expressing breast cancer patients. The model predicted HER2 heterogeneity and pCR with diagnostic accuracy of 97.4% and 83.9% in the test set, respectively. More specifically, the HER2 express status in breast carcinoma could be better predicted by a radiomics signature established from the mpMRI compared with a single-parametric signature. Huang et al. [43].showed that multi-parametric MRI-based radiomics combined with different machine learning approaches could be a promising method to predict the molecular subtype and AR expression of breast cancer non-invasively. In this study, the results verified the conclusion with the superior performance of prediction, in which the Rad score based on mpMRI images achieved higher AUC values of 0.927 and 0.826 than other single-parametric models (i.e., ADC-map, T2W1, and DCE-T1W1) in training (AUC: 0.816, 0.743 and 0.810) and validation sets (AUC: 0.719, 0.694 and 0.756), respectively.

Many studies have reported the association between histological grade, Ki-67 index, and HER2 status; HER2-positive expression has a relationship with high nuclear grade and ki-67 index [44–46]. In contrast, our study showed that only the ki-67 index and histological grade could be used as an independent predictor of HER2 expression. The ki-67as proliferation marker has been successfully used as a tool for clinical decision-making, specifically how it can be used to select the optimal treatment for each individual patient [47]. Tumor histological grade has previously been linked to HER2 status. HER2 expression has previously been found to correlate with a higher nuclear grade but not with tumor stage [45]. However, none of these studies developed a predictive model that included these predictors. Our study is the first to develop a nomogram to perform this prediction. Recently, the added value of the nomogram model based on radiomics signature and clinical factors for pathological outcome prediction [48–50] has been investigated by some studies. Our results showed the highest performance compared with existing work, which is another clinical contribution of this work. In this study, 11 optimal features were finally retained for modeling, including morphological, first-order, GLCM, GLDM, and GLSZM characteristics, which are predominantly involved in tumor heterogeneity. Based on tumor size and morphology, a significant correlation existed between the characteristics of shape_Maximum 2D Diameter, shape_Maximum 3D Diameter, shape_Sphericity, and the HER2 expression of breast carcinoma. Consistent with the previous report [51], the morphology and size of lesions vary with the different expressions of hormone receptors (HR). Much irregular and larger lesions were reported in the cases of HR-negative plus HER2-positive compared with the hormone HR-positive plus HER2-negative. In the present study, high kurtosis was found in most HER2-positive cases, which is consistent with the existing work. Fan et al. [52] constructed a predictive model for four molecular subtypes classification of breast carcinoma based on the DCE-T1W1 images and two clinical risk factors, revealing the potential of kurtosis as an independent predictor for classifying molecular subtypes of breast carcinoma. Previous studies have shown that texture analysis can provide information on tumor cellular and histological components [53, 54]. The SizeZoneNonUniformityNormalized texture feature measures the variability of zone volumes throughout the image. A higher value indicates more inhomogeneity among zone volumes in the image; thus, we concluded the irregular volume distribution of the HER2-positive group, which may be caused by the unequal growth rate of tumors in various directions. The texture features of glcm_Idmn, glcm_Dmn, and gldm_Dependence from wavelet-transformed images can reflect the homogeneity and consistency of tumor texture characteristics, conducive to the better exploration of intra-tumor heterogeneity and subtle differences in grey and texture level feature [55]. The DependenceEntropy texture feature measures the intensity differences in the neighborhood; the higher value represents a more disordered internal density of the tumor; hence, we found the complex heterogeneity existed in the HER2-positive texture patterns.

The mpMRI facilitates a comprehensive analysis of radiomics features; the established nomogram can fully combine the characteristics of different sequences. Nonetheless, there are several limitations to this study. Firstly, the limited sample size and all the cases were from one center. Therefore, additional validation of the constructed models on multicentric studies with a large study population is required. Second, to avoid confounding influences associated with the pathological type, the study only enrolled patients with IDC-NST, the most common form of breast carcinoma. Thirdly, we only used the traditional machine learning classification algorithms to explore the predefined radiomics features instead of the more informative and abstract features extracted by deep learning algorithms from MR images.

## Conclusions

This study showed that radiomics signature based on mpMRI could improve the discriminative performance of HER2 status prediction in patients with breast carcinoma. The nomogram with the radiomics signature and independent clinical risk factors is a key tool for noninvasive diagnosis.

## Supplementary Information

**Additional file 1: Table S1.** Statistically analysis of the final selected radiomics features based on mpMRI in the training and validation sets. **Table S2.** Statistically analysis of the six radiomcis classifiers and their corresponding nomograms in the validation cohort. **Figure S1.** Two typical HER2-positive and HER2-negative cases. (a) Original DCE-T1WI image of the HER2-positive sample. (b) The semi-automated segmentation of lesion on Deepwise. (c) Manual modification. (d) Lesion segmentation in T2WI. (e) Lesion segmentation in ADC-map. (f) The corresponding 3D ROI. (g-l) The corresponding images for the HER2-negative sample. **Figure S2.** ROC curves of T2WI model, ADC-map model, DCE-T1WI model, and Rad score in evaluating HER2 status of breast carcinoma. A. Training cohort; B. Validation cohort. **Figure S3.** Nomogram performances for predicting HER2 status of breast carcinoma in the Validation set. A. ROC curves of the six nomograms based on different radiomics classifiers. B. Calibration curve of the nomogram_RF. C. Decision curves of the nomogram_RF and Rad score.

## Data Availability

The datasets used and/or analyzed during the current study are available from the inside author on reasonable request. The study data can be made available publicly.

## Abbreviations

LASSO: Least absolute shrinkage and selection operator
RFE: Recursive feature elimination
Rad score: Radiomics score
ROC: Receiver operating characteristic
DCA: Decision curve analysis
IHC: Immunohistochemistry
FISH: Fluorescence in-situ hybridization
IDC: Invasive ductal carcinoma
T2WI: T2-weighted images
ADC-map: Apparent diffusion coefficient map
DCE-T1WI: Dynamic contrast-enhanced T1 weighted images
IDC-NST: Invasive breast ductal carcinoma of no special type
GLCM: Gray level co-occurrence matrix
GLRLM: Gray-level run-length matrix
GLSZM: Gray-level size zone matrix
GLDM: Gray-level dependence matrix
AUC: Area under the curve
ASCO: American Society of Clinical Oncology
CAP: College of American Pathologists
LR: Logistic regression
LDA: Linear discriminant analysis
SVM: Support vector machine
RF: Random forest
NB: Naive Bayesian
XGB: XGBoost
