# Supplementary material for: Development and validation of a clinicoradiomic nomogram to assess the HER2 status of patients with invasive ductal carcinoma
Source: BMC Cancer. 2022 Aug 10;22:872. doi: 10.1186/s12885-022-09967-6 (PMC9364617; doi:10.1186/s12885-022-09967-6)
Supplement: Supplementary file 1 — Additional file 1: Table S1. Statistically analysis of the final selected radiomics features based on mpMRI in the training and validation sets. Table S2. Statistically analysis of the six radiomcis classifiers and their corresponding nomograms in the validation cohort. Figure S1. Two typical HER2-positive and HER2-negative cases. (a) Original DCE-T1WI image of the HER2-positive sample. (b) The semi-automated segmentation of lesion on Deepwise. (c) Manual modification. (d) Lesion segmentation in T2WI. (e) Lesion segmentation in ADC-map. (f) The corresponding 3D ROI. (g-l) The corresponding images for the HER2-negative sample. Figure S2. ROC curves of T2WI model, ADC-map model, DCE-T1WI model, and Rad score in evaluating HER2 status of breast carcinoma. A. Training cohort; B. Validation cohort. Figure S3. Nomogram performances for predicting HER2 status of breast carcinoma in the Validation set. A. ROC curves of the six nomograms based on different radiomics classifiers. B. Calibration curve of the nomogram_RF. C. Decision curves of the nomogram_RF and Rad score. [file 12885_2022_9967_MOESM1_ESM.docx]

***Supplemental Material***

1. **Supplemental Tables**

**Table S1** Statistically analysis of the final selected radiomics features based on mpMRI in the training and validation sets

| **Characteristics** | **Training cohort** | **Validation cohort** | **p-value** |
| --- | --- | --- | --- |
| original_shape_Sphericity.1 | 0.73 ±0.07 | 0.73 ±0.07 | 0.541 |
| wavelet.HHH_firstorder_Kurtosis.1 | 7.06 ±5.78 | 7.01 ±4.20 | 0.944 |
| exponential_glszm_SizeZoneNonUniformityNormalized.1 | 0.41 ±0.19 | 0.37 ±0.16 | 0.097 |
| gradient_gldm_LargeDependenceEmphasis.2 | 13.14 ±6.59 | 14.58 ±9.24 | 0.212 |
| wavelet.LLH_gldm_DependenceVariance.2 | 3.86 ±2.46 | 3.80 ±2.93 | 0.867 |
| original_shape_Maximum2DDiameterRow.3 | 30.72 ±15.31 | 32.98 ±16.78 | 0.311 |
| original_shape_Maximum3DDiameter.3 | 37.63 ±20.01 | 42.40 ±21.93 | 0.102 |
| original_gldm_LargeDependenceHighGrayLevelEmphasis.3 | 8882.5 ±5069.0 | 9123.2 ±4236.1 | 0.717 |
| wavelet.LLH_glcm_Idmn.3 | 0.96 ±0.02 | 0.96 ±0.04 | 0.755 |
| wavelet.HLH_glcm_Idn.3 | 0.87 ±0.03 | 0.88 ±0.03 | 0.107 |
| lbp.3D.k_gldm_DependenceEntropy.3 | 4.49 ±0.39 | 4.50 ±0.49 | 0.937 |

**Table S2** Statistically analysis of the six radiomcis classifiers and their corresponding nomograms in the validation cohort

|  | **Training set** | | | **Validation set** | | |
| --- | --- | --- | --- | --- | --- | --- |
|  | **AUC（95%CI）** | | **p-value** | **AUC（95%CI）** | | **p-value** |
|  | **Radiomics classifier** | **Nomogram** |  | **Radiomics classifier** | **Nomogram** |  |
| LR | 0.887 (0.829-0.946) | 0.907 (0.857-0.957) | 0.375 | 0.810 (0.709-0.905) | 0.853 (0.771-0.935) | 0.100 |
| LDA | 0.884 (0.824-0.944) | 0.898 (0.845-0.952) | 0.366 | 0.801 (0.701-0.901) | 0.854 (0.772-0.935) | 0.100 |
| SVM | 0.887 (0.827-0.947) | 0.915 (0.864-0.968) | 0.236 | 0.840 (0.758-0.922) | 0.877 (0.801-0.953) | 0.123 |
| RF | 0.927 (0.876-0.978) | 0.945 (0.904-0.987) | 0.374 | 0.826 (0.738-0.914) | 0.868 (0.789-0.948) | 0.076 |
| NB | 0.885 (0.825-0.946) | 0.904 (0.852-0.956) | 0.308 | 0.788 (0.694-0.882) | 0.835 (0.751-0.919) | 0.086 |
| XGB | 0.890(0.931-0.949) | 0.908 (0.858-0.959) | 0.230 | 0.790(0.688-0.891) | 0.820 (0.728-0.913) | 0.308 |

1. **Supplemental Figures**

**
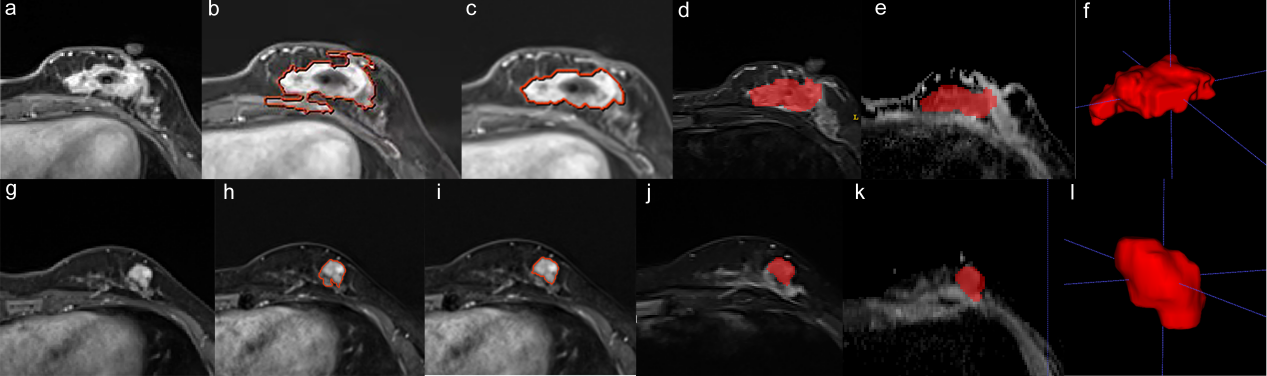
**

**Figure S1.** Two typical HER2-positive and HER2-negative cases. (a) Original DCE-T1WI image of the HER2-positive sample. (b) The semi-automated segmentation of lesion on Deepwise. (c) Manual modification. (d) Lesion segmentation in T2WI. (e) Lesion segmentation in ADC-map. (f) The corresponding 3D ROI. (g-l) The corresponding images for the HER2-negative sample.


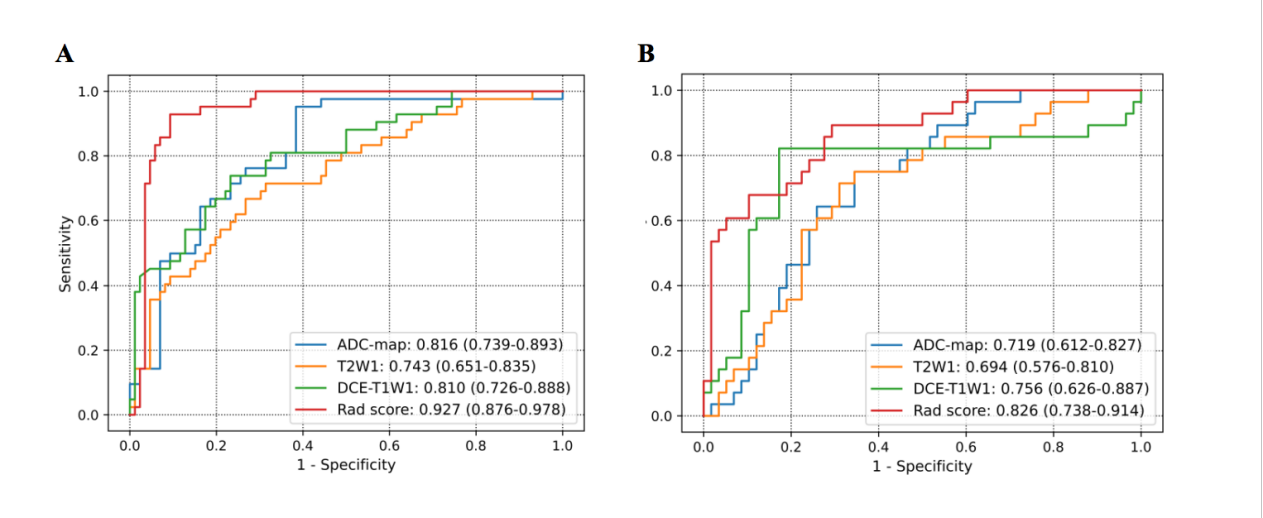


**Figure S2.** ROC curves of T2WI model, ADC-map model, DCE-T1WI model, and Rad score in evaluating HER2 status of breast carcinoma. A. Training cohort; B. Validation cohort


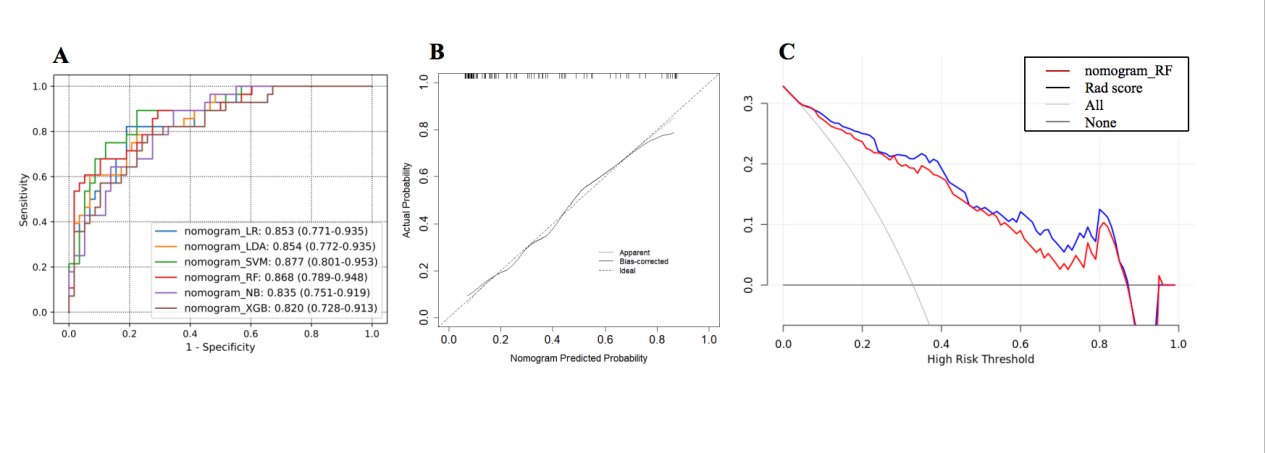


**Figure S3.** Nomogram performances for predicting HER2 status of breast carcinoma in the Validation set. A. ROC curves of the six nomograms based on different radiomics classifiers. B. Calibration curve of the nomogram_RF. C. Decision curves of the nomogram_RF and Rad score
